# Health-related quality of life in children with congenital vascular malformations

**DOI:** 10.1007/s00431-023-05166-y

**Published:** 2023-09-04

**Authors:** Frédérique C. M. Bouwman, Chris Verhaak, Ivo de Blaauw, Leo J. Schultze Kool, D. Maroeska W. M. te Loo, Iris A. L. M. van Rooij, Carine J. M. van der Vleuten, Sanne M. B. I. Botden, Bas H. Verhoeven

**Affiliations:** 1 Department of Pediatric Surgery, Radboudumc-Amalia Children’s Hospital, P.O. Box 9101, 6500 HB Nijmegen, the Netherlands; 2 Department of Radiology and Nuclear Medicine, Radboudumc, P.O. Box 9101, 6500 HB Nijmegen, the Netherlands; 3 Radboudumc Center of Expertise for Vascular Anomalies Hecovan, VASCERN VASCA European Reference Center, P.O. Box 9101, 6500 HB Nijmegen, the Netherlands; 4 Department of Medical Psychology, Radboudumc, Nijmegen, the Netherlands; 5 Department of Pediatric Hematology, Radboudumc-Amalia Children’s Hospital, Nijmegen, the Netherlands; 6 Department of Health Evidence, Radboudumc, Nijmegen, the Netherlands; 7 Department of Dermatology, Radboudumc, Nijmegen, the Netherlands

**Keywords:** Vascular malformations, Lymphatic malformations, Venous malformations, Arteriovenous malformations, Health-related quality of life, Sclerotherapy

## Abstract

A cross-sectional study was performed to evaluate health-related quality of life (HRQOL) in children with congenital vascular malformations (CVM) and to investigate factors associated with an impaired HRQOL. Children (2–17 years) with CVMs who visited the HECOVAN expertise center between 2016–2018 were included. The PedsQL 4.0 Generic Core Scales were used and a score ≥ 1.0 SD below the normative mean was defined as an impaired HRQOL. Factors associated with impairment were investigated using univariate and multivariate logistic regression analysis. The median overall HRQOL was 84.8/100 (n = 207; 41% boys, 59% girls; self-reported IQR 73.9–92.4 and parent-reported IQR 71.4–92.4). Patients aged 13–17 years reported significantly worse physical functioning than those aged 8–12 years (median 84.4, IQR 71.1–93.8 versus median 90.6, IQR 81.3–96.9; p = 0.02). Parents reported a significantly lower overall HRQOL than their children (median 80.4, IQR 70.7–90.8 versus median 85.9, IQR 76.1–92.4; p = 0.001). HRQOL was impaired in 25% of patients. Impairment occurred significantly more often in lower extremity CVMs (38%, p = 0.01) and multifocal CVMs (47%, p = 0.01) compared to CVMs in the head/neck region (13%). Other associated factors included invasive management (31% versus 14%; p = 0.01), age at first treatment ≤ 5 years (48% versus 25%; p = 0.02) and ongoing treatment (38% versus 18%; p = 0.004). After correction for other factors, significance remained for lower extremity CVMs and ongoing invasive treatment.

*Conclusions*: Overall median HRQOL was reasonable and not significantly different from the norm sample. Parental ratings were significantly lower than their children’s ratings. A quarter of the patients had an impaired HRQOL, which seemed to worsen with age. Independently associated factors included a lower extremity CVM and invasive management.

**Table:** 

**What is Known:** *• Congenital vascular malformations could affect health-related quality of life (HRQOL).* *• Studies on pediatric patients are limited and either very small or in combination with adult patient series.*	
**What is New:** *• This study raises awareness of an impaired HRQOL in 25% of pediatric patients with congenital vascular malformations.* *• Associated factors included a lower extremity CVM and invasive management.*

**What is Known:**

*• Congenital vascular malformations could affect health-related quality of life (HRQOL).*

*• Studies on pediatric patients are limited and either very small or in combination with adult patient series.*

**What is New:**

*• This study raises awareness of an impaired HRQOL in 25% of pediatric patients with congenital vascular malformations.*

*• Associated factors included a lower extremity CVM and invasive management.*

## Introduction

Congenital vascular malformations (CVMs) are rare developmental anomalies of the vascular system with an overall estimated prevalence of 1–1.5% [1–3]. Patients are often (and preferably) treated in multidisciplinary and interprofessional teams in so called centers of expertise, due to the rarity of the disease and possible difficulties and complications of treatment [3]. Both the disease, repetitive hospitalizations and treatment-related morbidity could affect health-related quality of life (HRQOL) [4]. HRQOL has been recognized as a core outcome measure in the international Outcome Measures for Vascular Malformations (OVAMA) project [5].

Nevertheless, there is a paucity of literature concerning HRQOL in children with CVMs. Physical impairment was reported in a study including both children and adults with venous malformations [6], whereas in a cohort of children with lymphatic malformations, values similar to norm samples were reported [7]. Factors associated with an impaired HRQOL were the number of sclerotherapy procedures, age at first treatment and location in the head and neck region [7]. However, it is difficult to generalize these results, because these studies were small and focused on a specific CVM subtype. The aim of this study was to evaluate HRQOL in a large cohort of children with a CVM and to investigate factors associated with an impaired HRQOL.

## Methods

### Study design

This cross-sectional study was performed at the HECOVAN expertise center for Hemangiomas and Congenital Vascular Malformations of the Radboudumc-Amalia Children’s Hospital in Nijmegen, the Netherlands. HECOVAN is recognized as a center of expertise within the European Reference Network. All children aged 2–17 years who were diagnosed with a CVM and visited the hospital between January 2016 and December 2018, were eligible for inclusion. The local ethical committee approved this study and written informed consent was obtained from all participating patients and/or parents as appropriate for the age of the child.

Medical records were reviewed for data on baseline, clinical and treatment characteristics. These data included sex, CVM subtype, CVM location, age at first treatment, type of management, and the number of procedures. The CVM subtypes included arteriovenous (AVM), venous (VM), lymphatic-venous (LVM; both lymphatic and venous components) and lymphatic malformations (LM). The anatomical location was divided into head and neck region, trunk, upper extremity, lower extremity and multifocal (multiple anatomical locations).

### Treatment of CVMs

Non-invasive management includes a wait-and-see approach or compression therapy. It is appropriate in case of CVMs that cause little or no complaints and if the risks of invasive management outweigh the risks and symptoms of the disease [8]. Invasive management includes sclerotherapy or embolization, which is usually the primary treatment of choice in this center [8–10]. Endovascular management can be combined with surgery, either by performing debulking in case of functional impairment or as the last step by removal of the residual mass. In addition, the detection of genetic mutations in CVMs revealed new targets for pharmaceutical treatment, such as the mTOR inhibitor sirolimus [11]. This cohort included patients treated both non-invasively and invasively. The patients who received sirolimus were all treated in an experimental setting at that time [12]; these patients were all treated with sclerotherapy, embolization, or surgery previously and therefore they were included in the invasively treated cohort.

### Questionnaire

The PedsQL 4.0 Generic Core Scales were used to evaluate HRQOL [13]. A self-report questionnaire was used for patients aged 8–17 years. The parent-proxy report was used for all patients aged 2–15 years. In the Netherlands, patients aged 16 and 17 years are allowed to give informed consent themselves without parental consent. Therefore, only the self-report was gathered in this age group.

The PedsQL questionnaire covers four key dimensions: physical, emotional, social, and school functioning and generates a score on a scale of 0–100 with higher scores indicating a better HRQOL [13, 14]. The total scale score (overall HRQOL), the physical health summary score (physical functioning subscale) and the psychosocial health summary score (emotional, social, and school functioning subscales) were calculated.

### Statistical analysis

Data were analyzed with IBM SPSS statistical software version 25. For categorical data, percentages were calculated, and data were compared using the Pearson Chi-square or Fisher’s exact test. For continuous data, medians with interquartile range (IQR) or means with standard deviations (SD) were calculated as appropriate. P-values less than 0.05 were considered significant.

Differences in PedsQL scores were evaluated between boys and girls, children and parents, non-invasive and invasive management, and the various categories of age, CVM location and subtype. In case of independent groups, the Kruskal–Wallis and Mann–Whitney U test were used. In case of two related groups (children and their parents), the Wilcoxon signed-rank test was used.

The PedsQL scores were compared with normative means of the general Dutch population [15]. These normative means were defined in a study by Engelen et al., which was aimed at collecting reference data by administering the PedsQL Generic Core Scale to Dutch school-aged children. Based on the distribution of data, the one sample t test or the one sample Wilcoxon signed rank test was used. Since these reference values were available from the age of 5 years, patients aged 2–4 years were excluded from these analyses. For the other age categories in the norm samples, the parent proxy-report was used for children aged 5–7 years and the child self-report for children aged 8–12 and 13–17 years [15]. A PedsQL score ≥ 1.0 SD below the normative mean was considered as an impaired HRQOL based on Varni et al. [14, 16, 17]. Patients with a normal and impaired HRQOL were compared using the Chi-square or Fisher’s exact test. Subsequently, a multivariable logistic regression analysis was conducted to assess the independent association of several factors. Variables were dichotomized based on the results of univariate analysis: age 5–12 versus 13–17, lower extremity versus other anatomical locations, and AVM and LM versus VM and LVM, and put together in the model. The logistic regression analysis was carried out with the binomial dependent variables of the PedsQL total scale score, physical functioning, and psychosocial functioning. In addition, this analysis was reproduced for the subgroup of patients treated invasively. Finally, a descriptive analysis was performed of the patients with a PedsQL score ≥ 2.0 SD below the normative means.

## Results

### Population

A total of 555 children were identified and approached to participate in this study. The included cohort consisted of 207 patients (207/555 = 37.3%). Table 1 shows the clinical characteristics. Most patients (n = 127; 61.4%) were treated invasively, with a median of two procedures per patient (IQR 1–4), although nine patients needed more than ten procedures. The number of patients treated with an mTOR inhibitor was ten.

**Table 1:** Descriptives

	**Number (%)**
**Total group**	207
**Sex**	
Boys	84 (40.6%)
Girls	123 (59.4%)
**Age category (years)**	
2–4	33 (15.9%)
5–7	39 (18.8%)
8–12	69 (33.3%)
13–17	66 (31.9%)
**CVM location**	
Head/neck	56 (27.1%)
Trunk	39 (18.8%)
Upper extremity	48 (23.2%)
Lower extremity	45 (21.7%)
Multifocal	19 (9.2%)
**CVM subtype**	
Arteriovenous	11 (5.3%)
Venous	117 (56.5%)
Lymphatic	47 (22.7%)
Lymphatic-venous	32 (15.5%)
**CVM associated with other anomaly**	
PROS / Klippel-Trenaunay syndrome	6 (2.9%)
PROS / CLOVES	2 (1.0%)
PROS / not otherwise specified	7 (3.4%)
Generalized lymphatic anomaly	1 (0.5%)
**Management**	
Duration of treatment expertise center	median 2.8 years, IQR 1.36–5.41
Non-invasive	80 (38.6%)
Invasive	127 (61.4%)
Sclerotherapy/embolization	119 (119/127 = 93.7%)
Surgery	27 (27/127 = 21.3%)
Nr of procedures	median 2, IQR 1–4
mTOR inhibitor	10 (10/127 = 7.9%)

*PROS* PIK3CA-related overgrowth spectrum, *CLOVES* congenital lipomatous overgrowth, vascular malformations, epidermal nevi, skeletal anomalies

### PedsQL scores

Compared to the norm sample, only the PedsQL scores in the physical domain in the age category 13–17 years differed significantly (Table 2). The median self-reported and parent-reported total scale scores were both 84.8 (IQR 73.9–92.4 and 71.4–92.4 respectively) (Tables 3 and 4).

**Table 2:** PedsQL scores compared to norm sample

**Age group**	**PedsQL subscale**	**Norm sample** **(mean ± SD)**	**Cut-off value for impaired HRQOL**	**CVM cohort** **(median, IQR)**	***p*-value**
**5–7 (PR)**	Total score	84.18 ± 8.96	75.22	87.0 (76.1–97.8)	0.35
	Physical domain	87.33 ± 10.25	77.08	90.6 (78.1–100)	0.60
	Psychosocial domain	82.50 ± 9.89	72.61	86.7 (78.3–96.7)	0.20
**8–12 (SR)**	Total score	82.11 ± 8.87	73.24	85.9 (77.2–92.4)	0.10
	Physical domain	84.87 ± 9.30	75.57	90.6 (81.3–96.9)	0.06
	Psychosocial domain	80.63 ± 10.31	70.32	86.7 (75.0–91.7)	0.16
**13–17 (SR)**	Total score	82.24 ± 9.15	73.09	82.6 (70.4–91.6)	0.47
	Physical domain	86.01 ± 9.77	76.24	84.4 (71.1–93.8)	0.04*
	Psychosocial domain	80.23 ± 10.18	70.05	81.7 (68.3–91.7)	0.44

One sample Wilcoxon signed rank test was used to compare the CVM cohort to the norm sample and 1 SD below the normative mean was used as cut-off value for the definition of an impaired HRQOL

*PR* parent-reported, *SR* self-reported, *SD* standard deviation, *HRQOL* health-related quality of life, *IQR* interquartile range, *CVM* congenital vascular malformation

*significant difference between patients aged 13-17 years and the norm sample aged 13-17 years

**Table 3:** PedsQL scores – parent-reported

**PedsQL subscale**	**Total group** **(n = 175)**	**Age 2–4** **(n = 33)**	**Age 5–7** **(n = 39)**	**Age 8–12** **(n = 67)**	**Age 13–15** **(n = 36)**	*p*-value*
Total score	84.8 (71.4–92.4)	85.7 (74.4–95.2)	87.0 (76.1–97.8)	80.4 (71.7–90.2)	78.3 (59.5–90.8)	0.05**
Physical domain	87.5 (75.0–96.9)	87.5 (78.1–98.4)	90.6 (78.1–100)	87.5 (78.1–96.9)	85.9 (66.4–93.8)	0.29
Psychosocial domain	82.7 (65.0–93.3)	84.6 (75.0–94.2)	86.7 (78.3–96.7)	78.3 (63.3–91.7)	76.7 (62.1–91.3)	0.04**

Medians and IQRs are presented

*Kruskal–Wallis test

**significant differences between patients aged 5–7 years and both 8–12 years and 13–15 years (Mann–Whitney U test)

**Table 4:** PedsQL scores – self-reported

**PedsQL subscale**	**Total group** **(n = 121)**	**Age 8–12** **(n = 59)**	**Age 13–17** **(n = 62)**	*p*-value*
Total score	84.8 (73.9–92.4)	85.9 (77.2–92.4)	82.6 (70.4–91.6)	0.16
Physical domain	87.5 (76.6–93.8)	90.6 (81.3–96.9)	84.4 (71.1–93.8)	0.02**
Psychosocial domain	83.3 (70.0–91.7)	86.7 (75.0–91.7)	81.7 (68.3–91.7)	0.42

Medians and IQRs are presented

^*^Mann–Whitney U test

^**^significant difference between patients aged 8–12 years and 13–17 years (Mann–Whitney U test)

#### Parental and children’s ratings

Tables 3 and 4 summarize the parent-reported and patient self-reported PedsQL scores. Post-hoc analyses of the parental ratings showed higher scores in the group of patients aged 5–7 years in the psychosocial domain and total scores compared to those aged 8–12 years and 13–15 years. Regarding the children’s ratings, only the physical domain was significantly different, with patients aged 13–17 years reporting worse physical functioning than those aged 8–12 years.

**Fig. 1:**
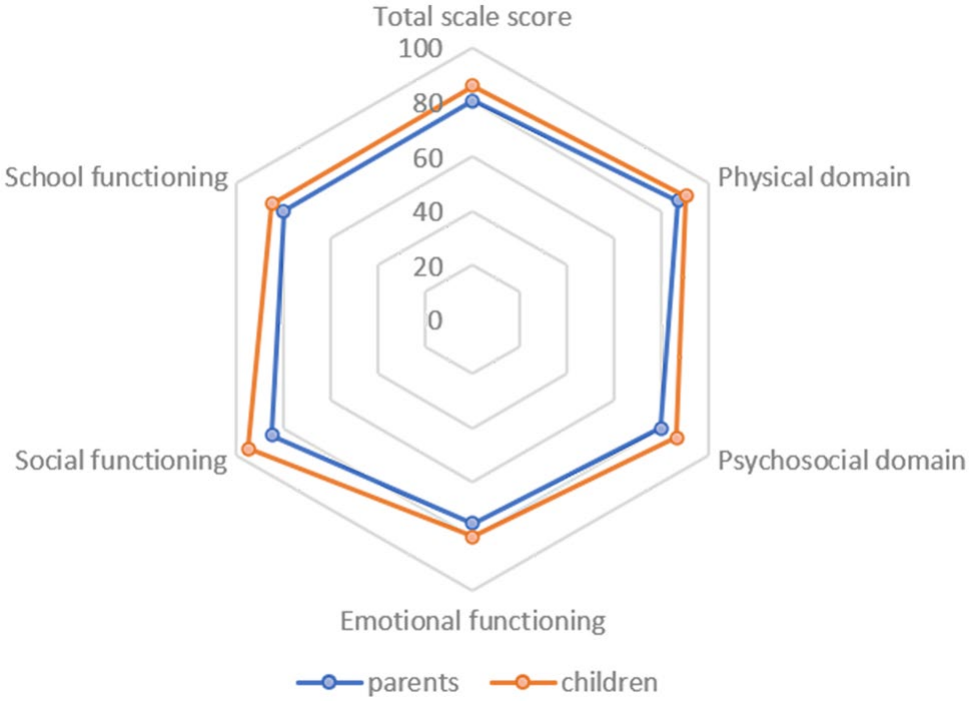
PedsQL scores compared between children and parents

To evaluate differences between parental and children’s ratings, a comparison was made in the age categories in which both parents and their children completed the questionnaires. Parents reported a significantly lower total scale score (parents: median 80.4, IQR 70.7–90.8; children: median 85.9, IQR 76.1–92.4; p = 0.001) and psychosocial functioning compared to their children (parents: median 80.0, IQR 64.2–91.7; children: median 86.7, IQR 75.0–92.5; p = 0.001) (Fig. 1). Similar results were found in the separate age categories.

#### Boys versus girls

Boys scored higher (median 93.8, IQR 46.9–100) than girls (median 84.4, IQR 37.5–100) on the self-reported physical functioning score (p = 0.005). Post-hoc analyses showed a significant difference in the age category of 13–17 years, whereas this was not the case in the norm sample.

#### Non-invasive versus invasive treatment

Parents reported significantly lower PedsQL scores in all domains for the children who were treated invasively (invasive: median total scale score 81.5, IQR 64.9–90.5; non-invasive: median total scale score 87.0, IQR 77.2–96.0; p = 0.004). The children’s ratings were also lower in case of invasive treatment, but not significantly different (invasive: median total scale score 83.7, IQR 72.8–92.4; non-invasive: median total scale score 87.0, IQR 75.8–94.8).

#### CVM subtypes and locations

No significant differences were found between the CVM subtypes, although the PedsQL scores were lowest for AVMs (Table 5). Table 6 shows the PedsQL scores in the various categories of CVM location. On the parental ratings, significant differences were found between a lower extremity CVM and a CVM in the head and neck region, trunk and upper extremity. On the children’s ratings, significant differences were found between either a lower extremity CVM or a CVM in the trunk and a CVM in the head and neck region.

**Table 5:** PedsQL scores compared between CVM subtypes

**Item**	**AVM**	**VM**	**LM**	**LVM**	*p*-value*
**All ages (PR)**	N = 9	N = 95	N = 42	N = 29	
Total scale score	72.8 (54.3–90.8)	84.8 (71.7–92.4)	85.8 (71.2–94.1)	85.9 (66.8–93.7)	0.58
Physical domain	81.3 (68.8–100)	87.5 (75.0–93.8)	87.5 (80.5–100)	90.6 (78.1–98.4)	0.89
Psychosocial domain	63.3 (54.2–85.8)	84.6 (68.3–93.3)	83.3 (63.4–94.2)	81.7 (66.7–93.3)	0.36
**All ages (SR)**	N = 9	N = 79	N = 18	N = 15	
Total scale score	82.6 (78.3–88.0)	84.8 (73.9–92.4)	87.0 (69.0–91.6)	85.9 (79.3–96.7)	0.62
Physical domain	84.4 (82.8–100)	84.4 (71.9–93.8)	85.9 (80.5–94.5)	90.6 (84.4–96.9)	0.36
Psychosocial domain	76.7 (75.0–87.5)	85.0 (70.0–93.3)	85.0 (62.9–90.4)	81.7 (76.7–96.7)	0.55

Medians and IQRs are presented; PR = parent-reported, SR = self-reported

*Kruskal–Wallis test

**Table 6:** PedsQL scores compared between CVM locations

**Item**	**Head/neck**	**Trunk**	**Upper extremity**	**Lower extremity**	**Multifocal**	*p*-value*
**All ages (PR)**	N = 52	N = 33	N = 38	N = 35	N = 17	
Total scale score	87.5 (73.9–92.4)	88.0 (78.9–97.1)	83.5 (76.9–91.6)	76.1 (65.2–90.2)	89.1 (52.4–95.4)	0.13
Physical domain	90.6 (84.4–96.9)	87.5 (79.7–100)	87.5 (80.5–93.8)	78.1 (62.5–90.6)	87.5 (50.0–96.9)	0.01**
Psychosocial domain	83.3 (63.4–91.7)	88.5 (76.9–96.7)	81.2 (71.3–92.1)	75.0 (61.7–93.3)	88.3 (59.2–95.4)	0.25
**All ages (SR)**	N = 33	N = 21	N = 27	N = 32	N = 8	
Total scale score	88.0 (79.9–95.1)	85.9 (72.3–91.3)	85.9 (72.8–93.5)	80.4 (68.5–88.9)	83.2 (68.2–94.6)	0.24
Physical domain	93.8 (84.4–98.4)	84.4 (68.8–92.2)	87.5 (75.0–93.8)	82.8 (68.8–87.5)	81.3 (67.2–98.4)	0.01***
Psychosocial domain	86.7 (75.8–93.3)	85.0 (73.3–92.5)	86.7 (66.7–96.7)	80.8 (68.8–89.6)	83.3 (64.6–98.8)	0.58

Medians and IQRs are presented; PR = parent-reported, SR = self-reported

*Kruskal–Wallis test

**significant differences between a lower extremity CVM and a CVM in the head/neck region (*p* = 0.001), trunk (*p* = 0.01) or upper extremity (p = 0.02) (Mann–Whitney U test)

***significant differences between either a lower extremity CVM or a CVM in the trunk and a CVM in the head/neck region (*p* = 0.001 and *p* = 0.02 respectively) (Mann–Whitney U test)

### Factors associated with an impaired HRQOL

An impaired HRQOL was found in 25% of the patients (≥ 1.0 SD below the normative mean, cut-off values in Table 2). In the subgroup of patients with CVMs associated with other anomalies, 11/16 patients were ≥ 5 years of age and the PedsQL scores could be compared with reference data. An impaired HRQOL was found in 5/11 (45%), with impaired physical functioning in 5/11 and impaired psychosocial functioning in 6/11.

Table 7 shows the distribution of patients with a normal and an impaired HRQOL, and the influence of clinical factors. Post-hoc analyses showed that patients aged 13–17 years significantly more often suffered from impaired physical functioning (33%) compared to patients aged 8–12 years (17%; p = 0.03). Patients with a lower extremity CVM or a multifocal CVM significantly more often had an impaired HRQOL compared to patients with a CVM in the head and neck region (38% versus 13%, p = 0.01, and 44% versus 13%, p = 0.01 respectively). In addition, significant differences were found in the physical domain between a CVM in the head and neck region and a CVM in the trunk (9% versus 28%, p = 0.05), and between an upper and lower extremity CVM (19% versus 43%, p = 0.03). Multivariable logistic regression analysis revealed that patients with a lower extremity CVM had a 3.2 times higher risk of having an impaired overall HRQOL compared to patients with a CVM elsewhere (95% CI 1.4–6.9), independent of sex, age, CVM subtype and type of management. The same was seen for the other domains, with a stronger association in the physical domain (OR 3.9, 95% CI 1.8–8.6) than in the psychosocial domain (OR 2.4, 95% CI 1.1–5.0). Invasive management was only identified as an independently associated factor in the physical domain (OR 2.7, 95% CI 1.1–6.9). Sex, age and CVM subtype did not independently contribute to HRQOL.

**Table 7:** Association between clinical characteristics and an impaired QoL

**Factor**	**Total scale score normal**	**Total scale score impaired**	*p*-value*	**Physical domain normal**	**Physical domain impaired**	*p*-value*	**Psychosocial domain** **normal**	**Psychosocial domain impaired**	*p*-value*
Overall (n = 174)	131 (75%)	43 (25%)		131 (75%)	43 (25%)		127 (73%)	47 (27%)	
**Sex**									
Boys (n = 67)	52 (78%)	15 (22%)	0.57	54 (81%)	13 (19%)	0.20	49 (73%)	18 (27%)	0.97
Girls (n = 107)	79 (74%)	28 (26%)		77 (72%)	30 (28%)		78 (73%)	29 (27%)	
**Age**									
5–7 (n = 39)	31 (79%)	8 (21%)	0.24	30 (77%)	9 (23%)	0.10	31 (79%)	8 (21%)	0.55
8–12 (n = 69)	55 (80%)	14 (20%)		57 (83%)	12 (17%)		50 (72%)	19 (28%)	
13–17 (n = 66)	45 (68%)	21 (32%)		44 (67%)	22 (33%)		46 (70%)	20 (30%)	
**CVM location**									
Head/neck (n = 47)	41 (87%)	6 (13%)	0.03**	43 (91%)	4 (9%)	0.003**	39 (83%)	8 (17%)	0.12
Trunk (n = 29)	22 (76%)	7 (24%)		21 (72%)	8 (28%)		22 (76%)	7 (24%)	
Upper extremity (n = 42)	34 (81%)	8 (19%)		34 (81%)	8 (19%)		32 (76%)	10 (24%)	
Lower extremity (n = 40)	25 (63%)	15 (38%)		23 (58%)	17 (43%)		25 (63%)	15 (38%)	
Multifocal (n = 16)	9 (56%)	7 (44%)		10 (63%)	6 (38%)		9 (56%)	7 (44%)	
**CVM subtype**									
Arteriovenous (n = 10)	8 (80%)	2 (20%)	0.38	8 (80%)	2 (20%)	0.79	8 (80%)	2 (20%)	0.45
Venous (n = 107)	82 (77%)	25 (23%)		78 (73%)	29 (27%)		78 (73%)	29 (27%)	
Lymphatic (n = 30)	19 (63%)	11 (37%)		23 (77%)	7 (23%)		19 (63%)	11 (37%)	
Lymphatic-venous (n = 27)	22 (81%)	5 (19%)		22 (81%)	5 (19%)		22 (81%)	5 (19%)	
**Management**									
Non-invasive (n = 66)	57 (86%)	9 (14%)	0.01***	58 (88%)	8 (12%)	0.003***	54 (82%)	12 (18%)	0.04***
Invasive (n = 108)	74 (69%)	34 (31%)		73 (68%)	35 (32%)		73 (68%)	35 (32%)	

Reference values available from age 5 (therefore total group for these analyses = 174), numbers represent absolute numbers and percentages

*Pearson Chi-square test

**significant differences in the distribution of normal and impaired HRQOL across the various CVM locations, results of post-hoc analysis reported in the Results section

***significant differences in the distribution of normal and impaired HRQOL between patients treated non-invasively and invasively, with impairment - both overall and in the physical and psychosocial subscales - more often found in patients treated invasively

In addition, subanalyses were performed concerning patients managed invasively. Children with an invasive procedure below the age of 5 years more often suffered from impaired psychosocial functioning compared to children who were older at first treatment (48% versus 26%; p = 0.04). Treatment status (finished or ongoing) was also identified as a significant contributing factor, with children receiving ongoing treatment more often present in the group of patients with an impaired HRQOL (38% versus 18%; p = 0.004). The age at initial presentation of the CVM and the number of procedures did not have a significant influence in univariate analysis. Multivariable logistic regression analysis of this invasively managed cohort showed that patients with a lower extremity CVM had a 4.9 times higher risk of having an impaired physical QOL compared to patients with a CVM elsewhere (95% CI 1.9–13.1), independent of sex, age, CVM subtype, age at first treatment, number of procedures and treatment status. This association remained in the overall HRQOL (OR 3.8, 95% CI 1.4–9.9), although it did not reach statistical significance as influencing factor in the psychosocial domain. In addition, ongoing treatment was identified as an independent influencing factor on physical functioning (OR 2.7, 95% CI 1.0–7.1). Age at first treatment was not an independent contributing factor.

A total scale score > 2 SD below the normative means was found in 23 children (23/174 = 13%). The cut-off values for the various age categories were 66.26 (5–7 years), 64.37 (8–12 years) and 63.94 (13–17 years). Physical functioning was more often affected (n = 24, 14%) than psychosocial functioning (n = 16, 9%). Most patients in this subgroup were treated invasively (19/23) and treatment started before the age of five years in 9/23. With regard to CVM location, more than half of the patients had a lower extremity (9/23) or multifocal (4/23) CVM.

## Discussion

This is the largest study about patient- and parent-reported HRQOL in children with congenital vascular malformations. The median total scale scores were reasonable with a score of 84.8 for both parent- and self-reported HRQOL. Further analyses showed that teenagers scored lower values on the physical domain and parents reported lower values mainly on the psychosocial domain. Although the median overall HRQOL was not significantly different from the norm sample, 25% of the patients in this cohort had an impaired HRQOL. Factors associated with an impaired HRQOL included a lower extremity CVM, age 13–17 years, invasive management, early start of treatment and ongoing treatment. Among these, a lower extremity CVM and ongoing invasive management were independent contributing factors.

Comparison with norm samples showed significantly lower physical functioning in patients aged 13–17 years. Teenage patients could be more bothered by or aware of their physical impairment than younger patients. Impaired physical functioning without significant psychosocial impairment has been described previously in pediatric patients with venous malformations, although the influence of age was not investigated [6]. In literature, comparisons between pediatric patients with congenital or chronic diseases and healthy controls showed significantly lower PedsQL scores in patients with congenital heart disease and osteogenesis imperfecta [18, 19]. On the other hand, no significantly different PedsQL scores were found in patients with esophageal atresia and Hirschsprungs disease [20, 21].

This study showed some interesting differences between parents and children rating HRQOL. In the direct comparison of questionnaires completed by children and their parents, parents reported a significantly lower overall HRQOL and psychosocial functioning. Although in the norm sample no comparisons were made between children and parents, another school-based study showed no significant differences in psychosocial health between children and their parents [22]. Furthermore, in case of invasive management, parents in this study rated the HRQOL significantly worse on all domains compared to non-invasive management, whereas no significant differences were found in the children’s ratings. Apparently, the perception of parents is different, or they suspect internalizing behavior. Although not previously investigated in children with CVMs, these findings are consistent with studies on children with chronic pain, type 1 diabetes, and pacemakers [23–25].

CVM location was identified as a significant influencing factor for impaired physical functioning, also after correction for other factors. A study including children with venous malformations also reported impaired physical functioning, although the cohort included patients with CVMs in the upper extremities, lower extremities and head/neck region, and the results were not specified for each CVM location [6]. In another study, head and neck lesions were associated with an impaired HRQOL compared to lesions elsewhere in the body [7]. This differs from the results presented here, showing that lower extremity and multifocal CVMs were associated with an impaired HRQOL. Previous studies on pediatric and adult patients identified a lower extremity CVM and muscle involvement as significant predictors of impaired physical functioning [26–28]. This is consistent with our findings and suggests that HRQOL is influenced by a combination of lesion characteristics.

In line with previous studies, no significant differences were found in HRQOL between the various CVM subtypes [27, 29, 30]. Nevertheless, the median scores in AVM patients were lowest, particularly in the psychosocial domain. This result was not unexpected, since AVMs are known to be more frequently associated with lesion-related complications, also in the cardiovascular system, and are generally more difficult to eradicate compared to other CVM subtypes. It could be argued that the non-significant differences between AVMs and other subtypes were due to the small cohort of AVM patients, and this CVM subtype was not always included in the abovementioned studies, probably also because of the rarity of the disease. On the other hand, it could be hypothesized from a clinical perspective that patients with other CVM subtypes are at risk of an impaired HRQOL, for example patients with a LM in the head and neck region or with excessive lymphatic fluid leakage, or patients with a VM with localized intravascular coagulopathy.

Patients who started treatment before the age of five years more frequently reported an impaired HRQOL. This could be due to disease severity, already requiring treatment at a younger age. The impact of early and invasive treatment itself could also play a role. It has been reported previously that early treatment is associated with poor treatment outcomes in neonates with a lymphatic malformation adjacent to the airway [31]. In contrary to this, Ghaffarpour et al. showed that young patients treated with sclerotherapy had a better HRQOL than older patients, although comparability is limited, since young patients were defined as < 16 years old when treatment started [7]. Surprisingly, this study did not find a significant influence of the number of procedures on HRQOL, unlike other studies [7, 32]. It could be that coping skills are adequate in at least part of the patients needing multiple procedures, resulting in non-significant differences between the groups.

Strengths of this study include the large pediatric cohort and the multidisciplinary approach. The cross-sectional design provides a reflection of reality; however, the differences between patients and the general population could be underestimated due to a subset of patients who have been treated successfully and thus might report an improved HRQOL. A possible weakness of this study is the heterogeneous cohort, and although subanalyses were possible, the results should be interpreted with caution due to relatively small subgroups.

This study substantially contributes to clinical practice by creating awareness of patients at risk of an impaired HRQOL, who may benefit from psychosocial or rehabilitation support, and it helps patients and doctors in their shared decision-making process whether or not to treat the CVM. This study also grounds and encourages future prospective studies, evaluating for example HRQOL over time, patient-reported weighted scores, and family impact. A disease-specific questionnaire, such as the recently validated OVAMA questionnaire, would complement the currently used generic questionnaires in future studies [33].

## Conclusion

An impaired HRQOL was found in 25% of the patients, although most patients had similar PedsQL scores compared to the norm sample. Factors independently contributing to the likelihood of an impaired HRQOL included a lower extremity CVM and invasive management. In addition, this study showed that parental ratings were significantly lower than their children’s ratings. These results underline the importance of addressing both parents and children during their visits. Ultimately, the goal of treatment should comprise both cure or stabilization of the CVM, as well as maintaining or promoting physical and psychosocial wellbeing.

## Data Availability

The datasets generated and analyzed during the current study are available from the corresponding author on reasonable request.

## Abbreviations

HRQOL: Health-related quality of life
CVM: Congenital vascular malformation
HECOVAN: Center of expertise for Hemangiomas and Congenital Vascular malformations in Nijmegen
OVAMA: Outcome Measures for Vascular Malformations
AVM: Arteriovenous malformation
VM: Venous malformation
LVM: Lymphatic-venous malformation
LM: Lymphatic malformation
IQR: Interquartile range
SD: Standard deviation
OR: Odds ratio
CI: Confidence interval
